# Experimental Access to Mode-Specific Coupling between Quantum Molecular Vibrations and Classical Bath Modes

**DOI:** 10.1021/acs.jpclett.3c01974

**Published:** 2023-09-20

**Authors:** Pankaj Seliya, Mischa Bonn, Maksim Grechko

**Affiliations:** Department of Molecular Spectroscopy, Max Planck Institute for Polymer Research, Ackermannweg 10, D-55128 Mainz, Germany

## Abstract

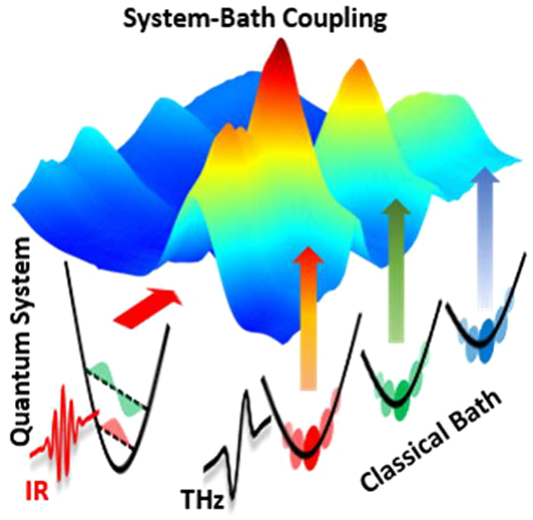

The interaction of quantum-mechanical systems with a fluctuating thermal environment (bath) is fundamental to molecular mechanics and energy transport/dissipation. Its complete picture requires mode-specific measurements of this interaction and an understanding of its nature. Here, we present a combined experimental and theoretical study providing detailed insights into the coupling between a high-frequency vibrational two-level system and thermally excited terahertz modes. Experimentally, two-dimensional terahertz-infrared-visible spectroscopy reports directly on the coupling between quantum oscillators represented by CH_3_ streching vibrations in liquid dimethyl sulfoxide and distinct low-frequency modes. Theoretically, we present a mixed quantum-classical formalism of the sample response to enable the simultaneous quantum description of high-frequency oscillators and a classical description of the bath. We derive the strength and nature of interaction and find different coupling between CH_3_ stretch and low-frequency modes. This general approach enables quantitative and mode-specific analysis of coupled quantum and classical dynamics in complex chemical systems.

Open quantum systems, two-level systems continuously exchanging energy with their environment, are ubiquitous in chemistry, ranging from molecules in solution to molecules at surfaces and at confinement. In these systems, high-frequency electronic excitations or molecular vibrations with energy quanta well-above the thermal energy *k*_B_*T*, with *k*_B_ the Boltzmann constant and *T* the temperature, are coupled to bath states in their environment. The latter are typically low-frequency intra- and intermolecular motions with energies comparable to *k*_B_*T* or smaller. Coupling to a bath is central to the evolution of quantum states giving rise to their energy fluctuations, dephasing, and thermalization. When there is also direct interaction between quantum systems, excitation transfer within quantum moiety is affected by bath dynamics, which causes fluctuations in their interaction strength.

Numerous theoretical approaches have been developed,^1^ in which the thermal bath is usually represented by its spectral density determined either phenomenologically^2−4^ or from numerical calculations.^5,6^ These considerations are typically based on significant approximations. Their benchmarking with detailed experimental data for real-world systems can help improve theories to the point that these could address cases relevant to chemistry.

Yet, experimental access to molecular open quantum systems is decidedly more challenging due to the limitations of techniques. Experimental access to the coupling is typically obtained indirectly using nonlinear spectroscopy, particularly transient absorption and two-dimensional electronic (2D ES) and infrared (2D IR) spectroscopies. These techniques can provide population relaxation and transfer rates, spatial reorientation, dephasing, and frequency fluctuations of a high-frequency quantum system.^7−15^ While insightful, such experiments are inherently limited concerning the details of the bath modes that shape the system dynamics, since there is intrinsically no mode specificity in the bath modes’ thermal excitation.

Detailed insights into the coupling between high-frequency quantum modes (HFMs) and low-frequency bath modes (LFMs) of molecular vibrations are enabled by the recently developed two-dimensional terahertz-infrared-visible (2D TIRV) spectroscopy.^16,17^ This technique allows for quantifying system-bath coupling strength in a mode-specific manner for both the LFM and HFM responses. However, deducing the characteristics of HFM-LFM coupling from 2D TIRV spectra requires rigorous simulations. Up to now, three different approaches have been reported in the literature for such simulations: using classical molecular dynamics (MD),^4^ ring-polymer MD (RPMD),^18,19^ and multimode Brownian oscillator (BO)^4,20^ models. MD has the advantage of explicitly considering intermolecular forces; thus, LFMs in this method have a clear physical origin. However, while classical approximation for low-frequency coordinates in MD calculations is reasonable, classical treatment of the quantum HFMs dynamics is a considerable simplification. RPMD accounts for quantum effects (proton delocalization) in the ground state of HFMs; however, its consideration of HFMs excitation remains essentially classical. In contrast, the BO model allows a quantum description of HFMs, but simplifies the bath degrees of freedom using a harmonic ansatz and postulating their static, homogeneous spectral density. An alternative approach, based on MD consideration of LFMs and quantum consideration of HFMs, can provide an understandable physical origin of their dynamics, allowing one to explicitly account for their microscopic inhomogeneity and correlations and thus improve accuracy and insights of the calculations.

A similar idea has been successfully used in computational absorption, Raman and 2DIR spectroscopies.^21−23^ In such a mixed quantum-classical formalism, the Hamiltonian of the quantum moiety depends parametrically on classical LFMs coordinates. The evolution of the latter is produced by (independently parametrized) MD simulation, which explicitly accounts for microscopic structural and dynamic inhomogeneity of the LFMs coordinates. Following these ideas, here we derive a quantum-classical sample response function measured with 2D TIRV spectroscopy. The obtained result is an analogue of the fully classical equilibrium-nonequilibrium formalism previously developed by Hasegawa and Tanimura.^24^

By combining this approach with state-of-the-art experimental data, we analyze a model molecular system, relatively simple CH_3_ stretch quantum oscillators in liquid dimethyl sulfoxide (DMSO). Studying coupling of a C–H stretch with bath degrees of freedom using conventional vibrational spectroscopy, e.g., spectral diffusion in 2D IR, is impeded by their typically close-to-homogeneous lineshapes. Symmetric and asymmetric CH_3_ stretching vibrations of DMSO molecules have frequencies of 2913 and 2996 cm^–1^, respectively (∼370 meV) (Figure 1c and Supporting Information Figure S1). We label these modes **s** and **a**, respectively. In the terahertz frequency range, DMSO shows sharp resonances at 333 and 383 cm^–1^ (∼45 meV) and a weak shoulder at 308 cm^–1^, which are assigned to intramolecular CH_3_–CH_3_ twist (*v*_23_), wagging (*v*_11_) and C–S–C bending (*v*_12_) vibrations, respectively.^25−27^ These modes are labeled by numbers 1, 2, and 3 (Figure 1c). We directly measure the coupling between CH_3_ stretch and terahertz modes of DMSO in a 2D TIRV experiment.

**Figure 1:**
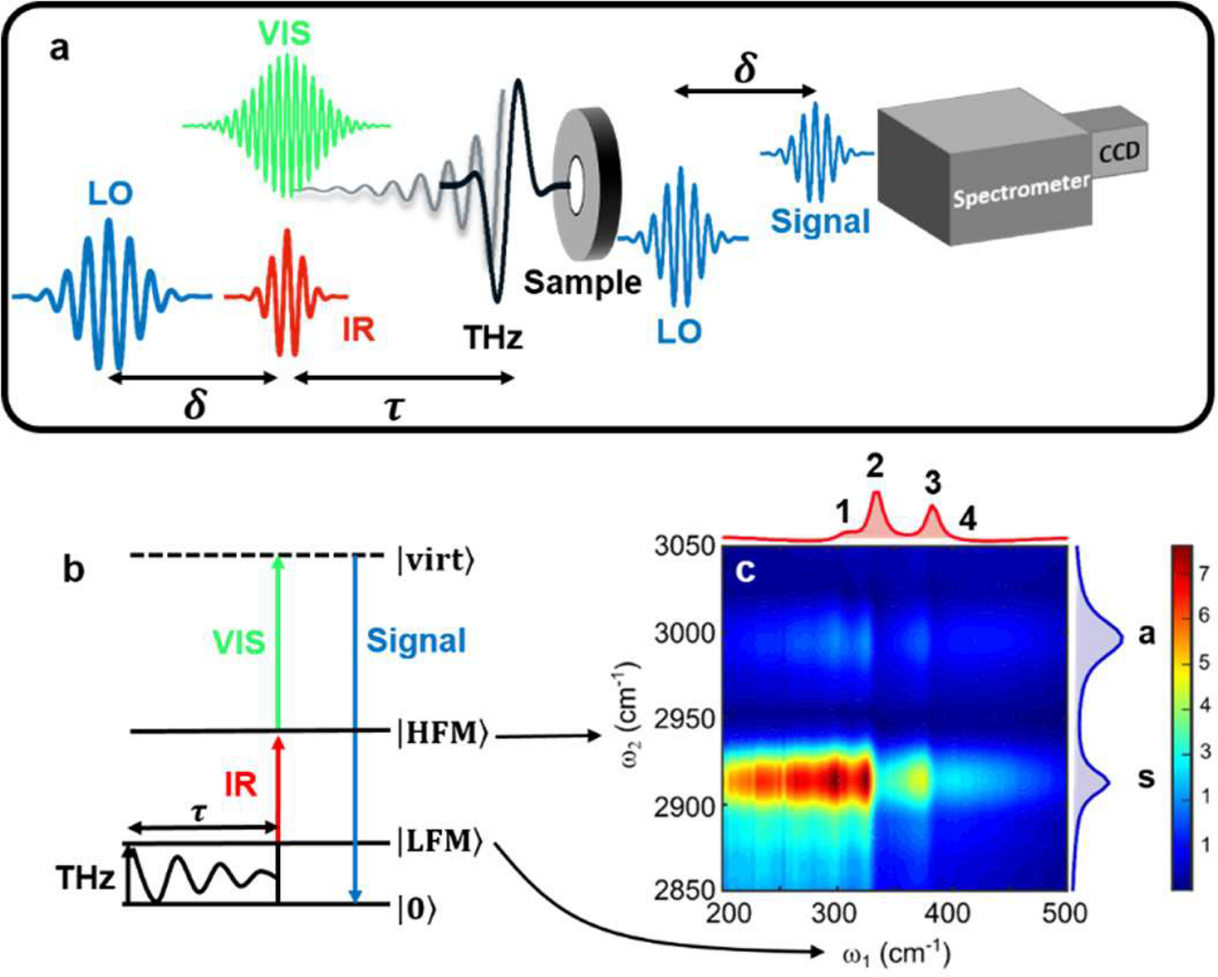
(a) Scheme of a 2D TIRV spectroscopy experiment. Gray shadowed line schematically shows low-frequency vibration excited in a sample by THz pulse. (b) Energy-level diagram of a 2D TIRV excitation pathway. (c) First quadrant of absolute-value 2D TIRV spectrum of liquid DMSO. Blue and red lines on the sides show mid- and far-infrared absorption spectra, respectively. The 2D spectrum reveals coupling between the two high-frequency modes (a and s) and the four low-frequency modes (labeled 1, 2, 3, 4).

Details of the 2D TIRV spectroscopy and treatment of the signals can be found in ref (17) (see also Experimental Methods section). In brief, a femtosecond, broadband terahertz (THz) pulse (Figure 1a) coherently excites the low-frequency modes in the sample (Figure 1b). A subsequent femtosecond mid-infrared (IR) and picosecond (narrowband) visible (VIS) pulse pair interrogates the sample. Simply put, HFM-LFM coupling is a prerequisite for the VIS+IR+THz sum or VIS+IR–THz difference frequency generation. We spectrally resolve the interference of signal and local oscillator (LO) pulses at different time delays τ between the terahertz and IR/VIS pulses using a spectrometer and charge-coupled device (CCD) detector. The terahertz spectral response (ω_1_-frequency) is obtained by Fourier transform of this interference along the τ-axis. Spectral resolution along the infrared axis (ω_2_-frequency) is obtained by correcting the spectrometer frequency for the known VIS frequency. Time delay δ between signal and LO is used in data processing to separate signals produced by VIS+IR+THz and VIS+IR–THz excitation pathways.^17^

Figure 1c shows the first quadrant of the absolute-value 2D TIRV spectrum |Γ_DMSO_ (ω_2_,ω_1_)| of neat liquid DMSO at 295 K. In the first quadrant, the signal originates from the sum-frequency mixing of the THz and IR fields. It contains pronounced peaks parallel to the ω_1_ axis at ω_2_ = 2913 and 2996 cm^–1^—frequencies of the CH_3_ stretching vibrations. This signal is broad over the ω_1_-frequency axis but contains sharp features around the ω_1_ frequencies of twist and wagging intramolecular motions. The shape of Γ_DMSO_ is affected by the spectral intensities and phases of the employed laser pulses. To eliminate this and extract the sample response function *S*_DMSO_^(3)^ (ω_2_,ω_1_), we use the 2D TIRV response of an ∼1 μm thick SiN_x_ membrane as a reference, as described elsewhere.^28^Figure 2 shows real and imaginary parts of the complex-valued *S*_DMSO_^(3)^ (ω_2_,ω_1_) in the first quadrant. This signal is generated by first and second interactions with THz and IR pulses, respectively.^28^ For each of the C–H stretching vibrational modes, the *S*_DMSO_^(3)^ response contains three peaks at ω_1_ ≈ 300, 326, and 376 cm^–1^. They reflect coupling with the intramolecular LFMs, which are pronounced in the absorption spectrum (Figure 1c, Supporting Information Figure S1). An additional peak centered at ω_1_ ≈ 410 cm^–1^ reflects strong coupling with LFM having weak absorption and being obscured in the absorption spectrum. The lineshapes of the 2D TIRV peaks closely resemble those of coupled oscillators in 2D IR spectra.^10,29^

**Figure 2:**
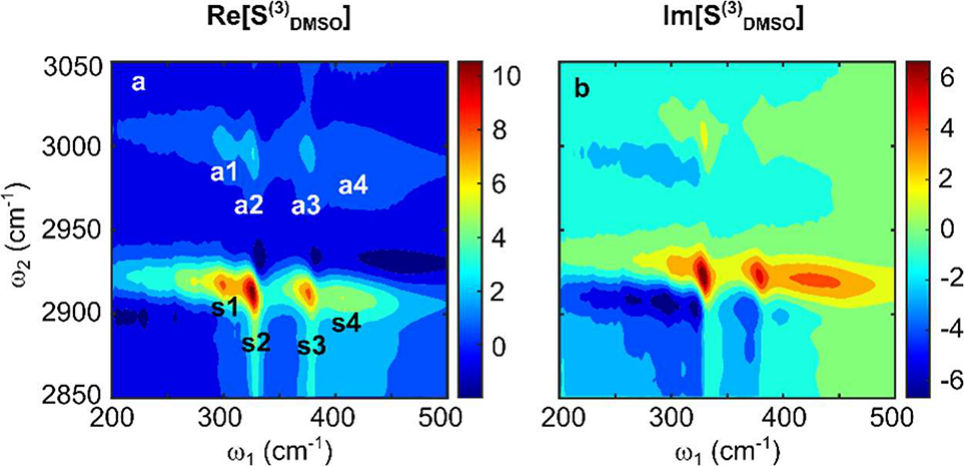
(a) Real and (b) imaginary part of the DMSO response function in the first quadrant measured by 2D TIRV spectroscopy. Peak labeling in (a) follows the notation in Figure 1c.

Obtaining quantitative insight into the interaction of CH_3_ stretch oscillators with their environment requires an accurate spectral analysis. The following equation gives the fully quantum 2D TIRV sample response function^9,30−32^
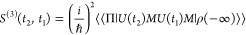
1where |ρ(−∞)⟩⟩ is the equilibrium density operator and *U* is the time evolution superoperator of the whole system, Π is the polarizability operator, and *M* is the dipole moment superoperator defined by the commutator (M = [μ,...]) with conventional dipole moment operator μ. The energy-level spacing of the terahertz modes is only ≈1.5–2 times larger than the thermal energy at room temperature (∼207 cm^–1^). Therefore, with reasonable accuracy, their dynamics can be considered classically.^33^ To this end, we use a partial Wigner representation of eq 1 and take the classical limit for LFMs coordinates (details are provided in the Supporting Information). The total *N* low-frequency degrees of freedom are represented by classical coordinate Q and momentum *P* (*Q* = {*Q*_*i*_ } and *P* = {*P*_*i*_}, *i* = 1...*N*). The total *K* high-frequency degrees of freedom are represented by quantum operators of coordinate *q̂* and momentum *p̂* (*q̂* = {*q̂*_*i*_} and *p̂* = {*p̂*_*i*_}, *i* = 1...*K*). We use a common approximation and neglect the influence of (excited) quantum degrees of freedom on the motion of classical degrees of freedom.^23,34,35^ The obtained mixed quantum-classical representation of the response function is given by
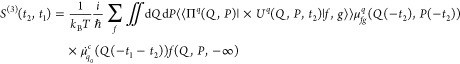
2

Here, *k*_B_, ℏ, and *T* are Boltzmann and reduced Planck constants and absolute temperature, respectively. The state of the classical subsystem is described by the probability density function *f*(*Q*,*P*,*t*), with *f*(*Q*,*P*,–∞) being an equilibrium distribution. The state of the quantum subsystem after photoexcitation by an IR field is described by the quantum density operator |*f*,*g*⟩⟩, where |*g*⟩ and |*f*⟩ are quantum ground and excited states, respectively. μ_*q*_0__^*c*^ (*Q*) is the classical dipole moment associated with low-frequency classical degrees of freedom *Q* and calculated at equilibrium position *q*_0_ of quantum degrees of freedom. In eq 2 we use time derivative μ̇_*q*_0__^*c*^ taken at time –*t*_1_ –*t*_2_, when classical coordinates are *Q*(−*t*_1_ –*t*_2_). Excitation of quantum modes by an IR pulse is described by matrix element μ_*fg*_^*q*^ of quantum dipole moment operator *μ*^*q*^ (*Q,P*) associated with the quantum subsystem. This quantum operator parametrically depends on the classical coordinates *Q* and *P*. Matrix element of *μ*^*q*^ (*Q,P*) is calculated for states |*f*⟩ and |*g*⟩ at time –*t*_2_. Following photoexcitation, the evolution of the quantum subsystem for a time period *t*_2_ is given by the quantum superoperator *U*^*q*^ (*Q,P,t*_2_) acting on state |*f,g*⟩⟩. Operator *U*^*q*^ (*Q,P,t*_2_) depends explicitly on time *t*_2_ and parametrically on classical coordinates *Q* and *P*. Π^*q*^ is the polarizability operator associated with the quantum subsystem, which depends parametrically on the classical coordinates *Q* and *P*.

In eq 2, the dependence of the transition dipole moment and polarizability operator on classical coordinates represents electrical coupling (anharmonicity) between HFMs and LFMs. Mechanical HFM-LFM coupling affects the evolution of quantum coherence |*f,g*⟩⟩ during the time period *t*_2_ and is embedded in the evolution superoperator *U*^*q*^. Quantum evolution superoperator *U*^*q*^ (*Q,P,t*) is a solution to the quantum Liouville equation of motion.
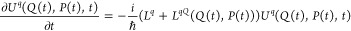
3

Here *L*^*q*^ is the Liouville superoperator of only quantum degrees of freedom. The Liouville superoperator *L*^*qQ*^ (*Q,P*) represents the interaction between quantum and classical coordinates—it acts on the state of the quantum subsystem and parametrically depends on classical coordinates. For the CH_3_ stretching vibrations of DMSO, we use the approximation of isolated resonances; i.e., we neglect intra- and intermolecular excitation transfer between them. In these limits, HFMs evolution reduces to the form^36,37^
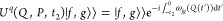
4where *ω*_*fg*_ is the instantaneous frequency of mode |*f*⟩, and this frequency depends on classical coordinates *Q*. Response function can, thus, be written in the following form.
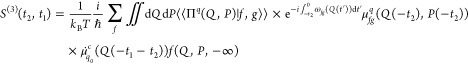
5

Instantaneous frequency *ω*_*fg*_ of the high-frequency coherence is affected by the interaction between the quantum system and the bath degrees of freedom. The latter is represented by two distinct types of modes. Bright modes with nonzero transition dipole moment interact with THz pulse and, thus, appear in the 2D TIRV spectrum explicitly at corresponding ω_1_-frequencies. Dark modes with zero transition dipole moments are not excited by the THz pulse and do not produce a signal. Still, they influence the spectrum by perturbing frequencies of HFMs and thus causing a broadening of spectral lines along the ω_2_-frequency axis.

Using eq 5, we simulate the response function of DMSO. To this end, we design a stochastic model by assigning an independent pair of high- and low-frequency modes for each resonance in the 2D spectrum. We label these pairs with indexes *mn* according to Figure 2a (*m* = *s,a*; *n* = 1,2,3,4). In this approximation, the LFM and HFM dynamics of all eight resonances are uncorrelated. Assuming small deviations *Q*_*n*_ of bright LFMs from their equilibrium positions, we consider the Taylor series of the frequency *ω*_*mn*_ (*Q*) of each CH_3_ mode up to the first-order term. In this approximation, *ω*_*mn*_ is linearly proportional to the LFM coordinate.
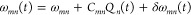
6

Coefficients *C*_*mn*_ are the coupling strengths for each HFM-LFM pair (*m, n*). The frequency fluctuation term δ*ω*_*mn*_ (*t*) represents the interaction of CH_3_ stretching vibrations with dark bath modes. Therefore, in our model, we explicitly account for the interaction between the quantum system and both bright and dark modes of environment.

In the experimental 2D TIRV spectrum of DMSO, the ω_2_-frequencies of the peaks match the frequencies of the CH_3_ stretching vibrations. Such signals are produced by a two-quantum transition upon interaction with the infrared laser pulse.^17^ In this case, the interaction with the visible pulse promotes a single-quantum transition and does not require any anharmonicity of the polarizability—i.e., we assume constant polarizability of CH_3_ stretching vibrations that equals its ensemble average value. We derive relative polarizabilities of symmetric and asymmetric modes from the Raman spectrum of DMSO (Figure S2 in Supporting Information). The integral Raman intensity of a mode is proportional to the square of its polarizability. Therefore, the polarizabilities of the two modes are ⟨⟨Π^*q*^ (*Q*)|*s,g*⟩⟩ = 1 a.u. and ⟨⟨Π^*q*^ (*Q*)|*a,g*⟩⟩ = 0.72 a.u. (here, a.u. stands for arbitrary units). The two-quantum transition upon infrared interaction requires mechanical anharmonicity and/or anharmonicity of the CH_3_ stretching transition dipole moment. We consider the response function *S*^(3)^ (*t*_2_,*t*_1_) for these two extreme cases separately.

First, we consider the anharmonicity of the transition dipole moment. In this case, in eq 6, the coefficient *C_mn_* =0. In the approximation of small deviation, the transition dipole moment *μ*_*mn*_ between the ground and first excited state of the *m*^*th*^ CH_3_ stretch oscillator depends linearly on the low-frequency coordinates (Taylor series truncated at the first order term), so that
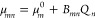
7where μ_*m*_^0^ is the transition dipole moment at the equilibrium position of *Q*, and the coefficients *B*_*mn*_ represent the strengths of HFM-LFM anharmonicity for each pair of vibrational modes. We derive μ^0^ of symmetric and asymmetric CH_3_ stretches from their integral intensities in the absorption spectrum. Assuming the transition dipole moment of symmetric stretch μ_*s*_^0^ = 1 a.u., we obtain the relative transition dipole moment of asymmetric stretch μ_*a*_^0^=1.48 a.u. (see Supporting Information). We note that in the case of electrical coupling μ_*m*_^0^ does not contribute to 2D TIRV signal generation. However, for the mechanical coupling considered below, the constant transition dipole influences the relative intensities of symmetric and asymmetric CH_3_ stretching vibrations.

The dipole moment of LFMs is linearly proportional to the coordinate *Q* for small deviations. Thus, the total dipole moment of low-frequency degrees of freedom is given by
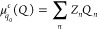
8

Coefficients *Z*_*n*_ represents the partial charges associated with the corresponding LFMs.

We use a harmonic model of the LFMs of DMSO because of their narrow line width and Lorentzian lineshapes. However, we emphasize that eqs 2 and 5 can be used for any kind of motion of a classical subsystem. We simulate trajectories of harmonic LFMs assuming an amplitude *Q*_*n*_^0^, a random initial phase *φ*_*mn*_, and frequency fluctuation δΩ_*mn*_ (*t*) of each mode.
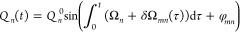
9

Thus, the time-dependent dipole moment of LFMs has the form
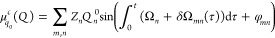
10

The product *Z*_*n*_*Q*_*n*_^0^ is the transition dipole moment of a mode and can be obtained from the absorption spectrum (Supporting Information Figures S1 & S3). The integral absorbance of a vibration is proportional to (*Z*_*n*_*Q*_*n*_^0^)^2^. Assuming *Z*_1_*Q*_1_^0^ = 1 a.u., relative transition dipoles are 3.69 and 3 a.u. for *n* = 2 and 3, respectively (see Supporting Information). For the mode *n* = 4, which is weak in the absorption spectrum, we assume a transition dipole moment of 1 a.u. In the Supporting Information, we demonstrate that this assumption is consistent with the linear absorption spectrum (Figure S4).

Realization of *Q*(*t*) and *ω*_*mn*_(*t*) generates a trajectory. To perform averaging over an ensemble of classical coordinates (∬d*Q*d*P*...), we calculate the expression below the integral in eq 5 for time intervals of *t*_1_ = 0···3000 fs and *t*_2_ = 0···1500 fs in time steps of Δ*t* = 15 fs for 2 × 10^6^ trajectories. To properly sample CH_3_ stretch oscillations, we decreased their frequency by 2912 cm^–1^. This offset is then added to the ω_2_-frequency produced in the calculation. At every time step, we assume a uniform probability distribution for frequency fluctuations δΩ_*mn*_ (*t*) and *δω*_*mn*_(*t*) and a phase change given by δΩ_*mn*_ (*t*)Δ*t* and δω_*mn*_ (*t*)Δ*t*, respectively. Averaging the responses generated by the trajectories produces time-domain response function *S*^(3)^ (*t*_2_,*t*_1_). The 2D spectrum is obtained by two-dimensional Fourier transformation of the calculated *S*^(3)^ (*t*_2_,*t*_1_).

The simulated spectrum reproduces the experiment reasonably well (Figure 3a,b) with values of the parameters *B*_*mn*_, Ω_*n*_, *ω*_*mn*_, and the range of frequency fluctuations *δΩ*_*mn*_ and *δω*_*mn*_ listed in Table I (difference spectrum is shown in Figure S5 in Supporting Information). Still, there is a subtle but noticeable discrepancy in lineshapes, which is most pronounced in the upper part of the s3 resonance. In this spectral range of ω_1_ = 315–405 cm^–1^, ω_2_ = 2915–2947 cm^–1^ the slopes of contours are significantly different in both real and imaginary parts of the spectra (Figure S6 in Supporting Information).

**Figure 3:**
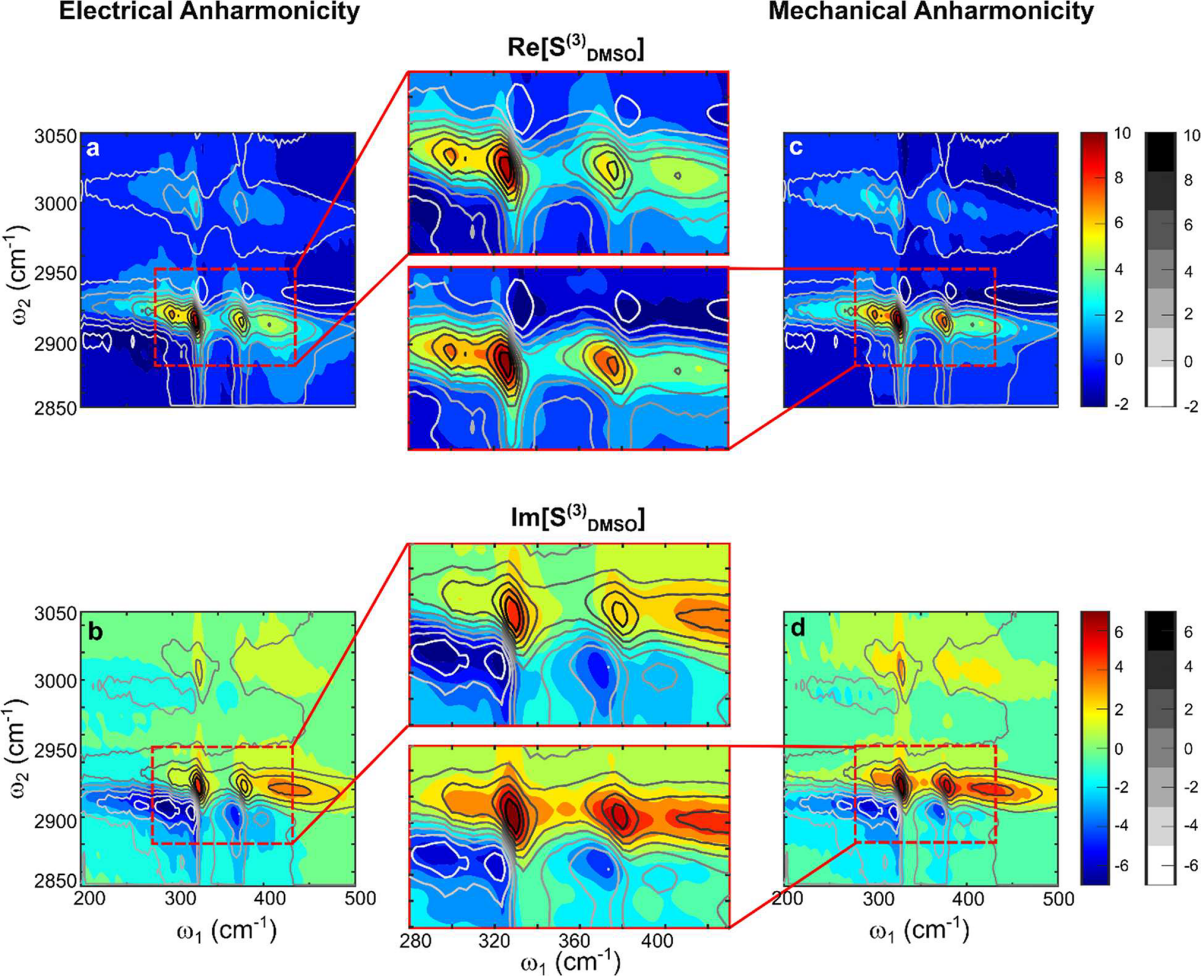
Comparison of theoretical and experimental spectra of DMSO. Colored contours show real (a, c) and imaginary (b, d) parts of the 2D TIRV spectrum calculated for the case of electrical (a, b) and mechanical (c, d) coupling. The corresponding color bars are shown on the right. Line contours show the real and imaginary parts of the measured spectrum. The corresponding grayscale bar is shown on the right. Note the better agreement with the mechanical anharmonicity model.

**Table I:** Parameters of the DMSO Model with Electrical/Mechanical HFM-LFM Coupling Shown in Figure 3a,b/c,d

mn	Ω_*n*_ (cm^–1^)	δΩ_*mn*_ (cm^–1^)	*ω*_*mn*_ (cm^–1^)	δ*ω*_*mn*_ (cm^–1^)	*B_mn_* (a.u.)/*C_mn_* (cm^–1^/*Q*_*n*_^0^)
**s**1	300/300	±210/ ±195	2915/2918	±130/ ±130	26/109
**s**2	326/326	±107/ ±107	2911/2912	±152/ ±152	2.7/21
**s**3	372/375	±141/ ±141	2912/2913	±169/ ±104	3.8/20
**s**4	406/412	±238/ ±271	2914/2916	±165/ ±54	30/178
**a**1	300/300	±210/ ±195	3000/3000	±133/ ±173	4.9/51
**a**2	326/326	±107/ ±107	2994/2994	±165/ ±228	1.3/10
**a**3	372/375	±141/ ±141	2994/2995	±152/ ±184	1.2/9
**a**4	406/412	±238/ ±271	2997/2998	±169/ ±141	17/122

In the second limit, we consider only the mechanical anharmonicity of the CH_3_ stretching vibrations. Similar to the first case, coupling with dark bath degrees of freedom causes the fluctuation *δω*_*mn*_(*t*) of the CH_3_ stretch frequencies. Besides that, mechanical coupling with bright LFMs produces additional perturbation of CH_3_ frequencies—i.e., in eq 6, coefficient *C*_*mn*_ ≠ 0.. Polarizabilities and transition dipole moments of CH_3_ modes are constant with the relative values discussed above (in eq 7, coefficient *B*_*mn*_ = 0).

Figure 3c,d shows excellent agreement between this model and the experiment (Figure S5 in Supporting Information shows the difference spectrum), with model parameters listed in Table I. The lineshapes match exceptionally well for both the real and imaginary parts of the response (see also Figure S6 in Supporting Information). Note that lineshapes produced by mechanical and electrical anharmonicities are qualitatively different, and this difference is rooted in the correlation of frequency fluctuations. In the computational model, HFMs and LFMs of DMSO are always broadened homogeneously, and their individual lineshapes are given by Lorentzian functions, in agreement with the absorption spectrum (Figure S3 in Supporting Information). With electrical HFM-LFM anharmonicity, the frequency fluctuations of CH_3_ stretching vibrations and bright LFMs are not correlated. In this case, a single resonance in the 2D TIRV spectrum has line shape symmetric with respect to its center (Figure 4a,b). This line shape is identical to that of a homogeneously broadened vibrational resonance with uncorrelated frequency fluctuations in 2D IR spectra.^10,29^ The lack of frequency fluctuation correlations results in a symmetric Lorentzian profile of a vertical cut taken through the resonance (Figure S7 in Supporting Information),^10^ at odds with the experimental line shape. In contrast, the correlation of frequency fluctuations produced by mechanical coupling causes asymmetry in the line shape of individual 2D TIRV peaks (Figure 4c,d, Figure S7 in Supporting Information). This results in an asymmetry in the overall spectrum in Figure 3 (see also Figure S6 in Supporting Information), in agreement with experimental line shape.

**Figure 4:**
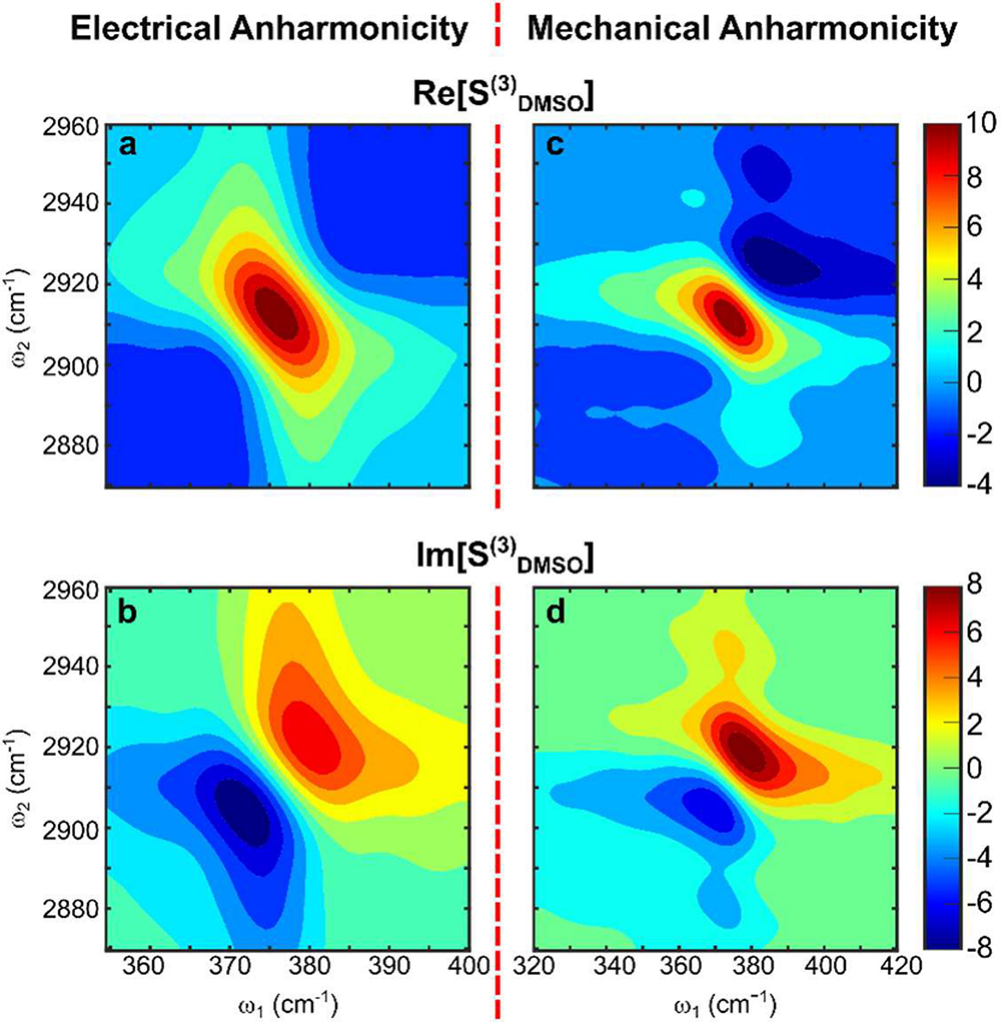
Comparison of single resonance line shape in 2D TIRV spectrum calculated assuming electrical (a,b) and mechanical (c,d) anharmonicity.

The 2D TIRV spectra thus show that the interaction between CH_3_ stretching vibrations and intramolecular LFMs of DMSO is dominated by mechanical coupling. This is plausible because of the modulation of the CH_3_ frequency caused by molecule distortion upon low-frequency motions. The parameters in Table I indicate similar coupling strength with intramolecular twist and wagging vibrations and about five times stronger coupling with the C–S–C bending mode. The *n* = 4 mode is not pronounced in the absorption spectrum, which hinders the unambiguous quantification of its transition dipole moment. However, its intense signal in the 2D TIRV spectrum demonstrates a particularly strong coupling with the CH_3_ stretching vibrations. Notably, the coupling is about two times stronger for the symmetric than the asymmetric stretch mode of the CH_3_ group.

Our results demonstrate that for isolated vibrational quantum modes, such as the C–H stretch, their coupling to the bath can be derived from experimental data using eq 5 and a stochastic model. In a more detailed description, the time dependence of coordinates *Q* and *P* in eq 2 is given by the classical Hamilton’s equations of motion
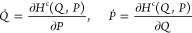
11where *H*^c^(*Q*,*P*) is Hamiltonian of a classical subsystem. Thus, in an exact approach, *Q*(*t*) and *P*(*t*) can be obtained from equilibrium classical MD simulations. For a given trajectory of classical coordinates, one can use numerical integration of the Schrodinger equation (NISE)^38,39^ to calculate the evolution of the quantum subsystem during a time period *t*_2_ (given by *U*^*q*^(*Q*,*P*,*t*_2_)|*f*,*g*⟩⟩ in eq 2). Averaging the term
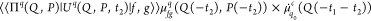
calculated for multiple MD trajectories provides an ensemble average over classical coordinates, which is represented in eq 2 by integral ∬d*Q*d*P*...*f*(*Q*,*P*,–∞). Such exact consideration of coupled quantum and classical dynamics can be particularly critical for systems with interacting quantum oscillators, like the O–H stretch in water and the C=O stretch in peptides—where the dependence of interaction strength on classical coordinates needs to be accounted for explicitly.

In summary, in this work, we use 2D TIRV spectroscopy experiments together with a novel quantum-classical description of sample response to directly measure and quantify the mode-specific coupling between quantum vibrational states and their classical environment. Using the example of CH_3_ stretching vibrations of liquid DMSO, we demonstrate that seemingly simple homogeneous energy broadening of a quantum oscillator can involve unequal interaction strengths with different thermally excited classical bath modes. The high quality of the experimental data and their excellent agreement with the theoretical model allow for determining a predominantly mechanical mechanism of HFM-LFM interaction; i.e., fluctuations of bath modes affect the potential of C–H covalent bonds. This coupling in the DMSO molecule can influence the reaction coordinate and facilitate intramolecular energy redistribution in several chemical reactions that involve breaking the C–H bonds. The developed quantum-classical description of sample response enables simultaneous explicit quantum consideration of high-frequency and classical consideration of thermally excited low-frequency vibrational dynamics. This formalism may especially benefit the investigation of excitonically delocalized quantum vibrations in molecular solids, liquids, and biomolecules, where inhomogeneity and correlations of quantum and classical subsystems can be critical.

## Experimental Methods

We use a femtosecond amplified Ti:sapphire laser system (Astrella, Coherent) with a repetition rate of 1 kHz, central wavelength of 800 nm, and full width at half-maximum (fwhm) of ∼60 nm. The output of the laser is split into three beams to generate broad-band terahertz (THz), broad-band infrared (IR), and narrow-band visible (VIS) pulses. THz pulse is produced from a laser beam with an energy of about 1.1 mJ/pulse by two-color mixing in air plasma.^40^ IR pulse is produced using commercial TOPAS (traveling wave optical parametric amplification of superfluorescence) coupled to NDFG (noncollinear difference frequency generation) unit. The TOPAS is pumped by a laser beam with ≈1 mJ/pulse energy. The VIS pulse (fwhm ≈ 10 cm^–1^) is produced using a 4f pulse shaper from a laser beam with ≈0.8 mJ/pulse energy. The local oscillator (LO) pulse is generated in a displaced Sagnac interferometer by the sum frequency mixing of IR and VIS fields in a type 1 BBO crystal (10 μm thickness, Newlight Photonics Inc.). All of the pulses are focused and overlapped at the sample to generate the third-order signal. After the sample, the signal and LO are collimated and directed to a spectrometer (SpectraPro HRS-300, Princeton Instruments) equipped with an electron-multiplying charge-coupled device (EMCCD) detector (Newton 971, Andor). We use parallel polarization of THz, IR, VIS, and signal fields. Energies of IR and VIS pulses at the sample are about 1.5 and 4.5 μJ/pulse, respectively. More details on the experimental setup, methods of data acquisition, and processing are provided elsewhere.^17^

Time domain data are acquired by varying the time delay between THz and the IR/VIS pulse pair in steps of 10 fs using a motorized delay stage (V-551.2B, Physik Instrumente (PI)). The total time intervals of scans for DMSO and SiN_*x*_ are 7.33 and 1.33 ps, respectively, with the same start position. It takes around 25 (5) min to acquire one spectrum of DMSO (SiN_x_).

The front window of the DMSO sample cell is a 50 nm thick low-stress SiN_x_ membrane (NX5050A, Norcada) with 0.5 mm × 0.5 mm aperture (in 5 mm × 5 mm, 200 μm thick silicon frame). The back window is 2 mm thick CaF_2_ window. Front and back windows are separated by a 1 mm thick Viton O-ring.

## Supporting Information Available

Derivation of the response functions and supplemental figures (PDF)
Transparent Peer Review report available (PDF)
