# Supplementary material for: Experimental Access to Mode-Specific Coupling between Quantum Molecular Vibrations and Classical Bath Modes
Source: J Phys Chem Lett. 2023 Sep 20;14(38):8630–7. doi: 10.1021/acs.jpclett.3c01974 (PMC10544034; doi:10.1021/acs.jpclett.3c01974)
Supplement: Supplementary file 1 — jz3c01974_si_001.pdf [file jz3c01974_si_001.pdf]

Supporting Information for

Experimental Access to Mode-Specific Coupling between Quantum Molecular Vibrations and Classical Bath Modes

Pankaj Seliya[1], Mischa Bonn[1], and Maksim Grechko[1*]

1. Department of Molecular Spectroscopy, Max Planck Institute for Polymer Research, Ackermannweg 10, D-55128, Mainz, Germany

*** Corresponding author**

Email: grechko@mpip-mainz.mpg.de

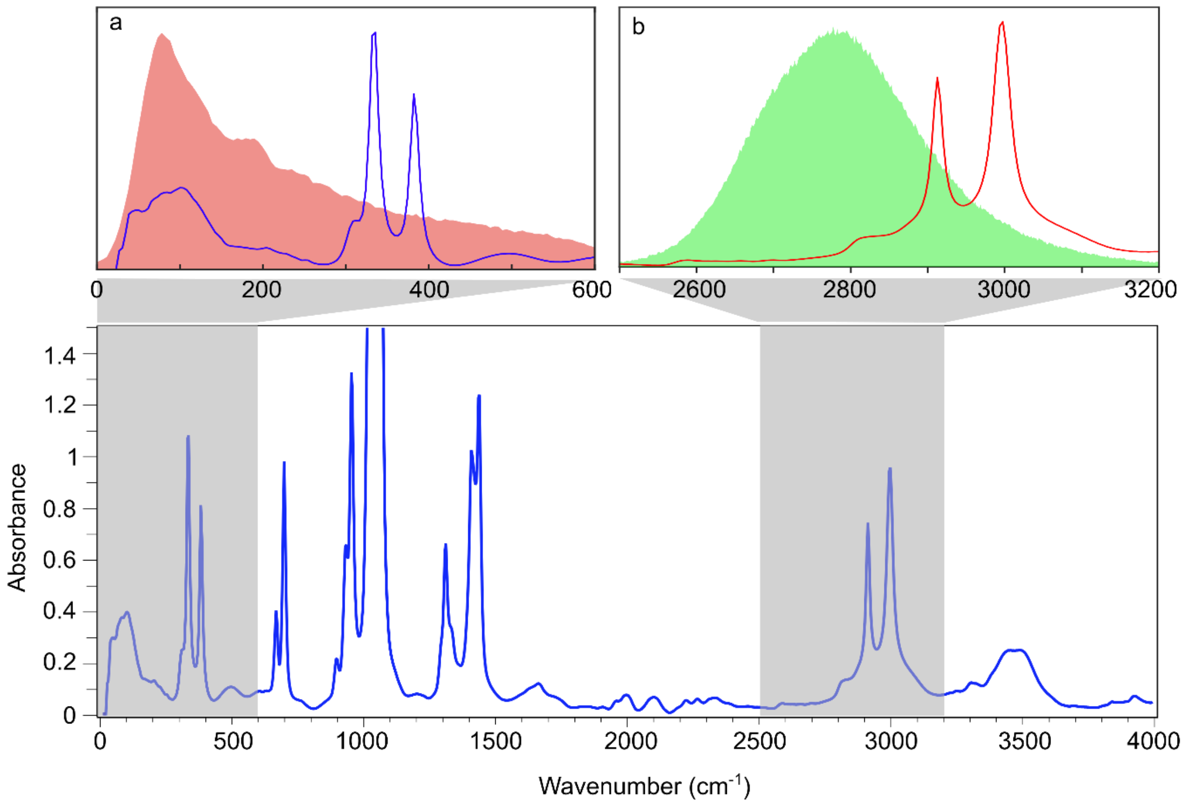

FIG. S1. The infrared absorption spectrum of DMSO, zoomed in at the relevant (a) low- and (b) high-frequency regions. Intensity spectra of THz and IR laser pulses are shown in red (a) and green (b), respectively.

Mixed quantum-classical formalism of response function

A. Mixed quantum-classical equation of motion

We consider a quantum system having multiple degrees of freedom. The state of the system is described by the density matrix operator $|\rho\rangle\rangle$, and its evolution is described by the Liouville equation

$$\frac{\partial|\rho(t)\rangle\rangle}{\partial t} = -\frac{i}{\hbar}L|\rho\rangle\rangle = -\frac{i}{\hbar}[\hat{H},\hat{\rho}], \tag{S1}$$

where L is Liouville superoperator and $\hat{H}$ is Hamiltonian operator. The accent $\hat{x}$ denotes a quantum operator in Hilbert space.

In the full system, we distinguish two groups of coordinates represented by the corresponding (multidimensional) operators $\hat{q}$ and $\hat{Q}$. Total Hamiltonian of the system is then composed of three terms

$$\hat{H} = \hat{H}^q + \hat{H}^Q + \hat{H}^{qQ}. \tag{S2}$$

The first ($\hat{H}^q$) and second ($\hat{H}^Q$) terms are Hamiltonians of the $\hat{q}$ and $\hat{Q}$ subsystems, respectively. The last term $\hat{H}^{qQ}$ describes the interaction between the two subsystems.

Partial Wigner representation of the Liouville equation in coordinates (Q,P) of the subsystem $\hat{Q}$ is given by:

$$\frac{\partial\langle\langle PQ|\rho(t)\rangle\rangle}{\partial t} = -\frac{i}{\hbar}\left([\hat{H}^q,\hat{\rho}]_{PQ} + [\hat{H}^Q,\hat{\rho}]_{PQ} + [\hat{H}^{qQ},\hat{\rho}]_{PQ}\right), \tag{S3a}$$

where

$$[\hat{H}^q,\hat{\rho}]_{PQ} = \left\langle\langle PQ\middle|[\hat{H}^q,\hat{\rho}]\rangle\right\rangle, \tag{S3b}$$

$$[\hat{H}^Q,\hat{\rho}]_{PQ} = \left\langle\langle PQ\middle|[\hat{H}^Q,\hat{\rho}]\rangle\right\rangle, \tag{S3c}$$

$$[\hat{H}^{qQ},\hat{\rho}]_{PQ} = \left\langle\langle PQ\middle|[\hat{H}^{qQ},\hat{\rho}]\rangle\right\rangle \tag{S3d}$$

We assume that total density matrix of the system is given by the product of the density matrixes of the two subsystems:

$$\hat{\rho} = \hat{\rho}^q\hat{\rho}^Q \tag{S4}$$

Thus, the first term on the right-hand side of Eq. S3a

$$\begin{aligned}[\hat{H}^q,\hat{\rho}]_{PQ} &= \left(\hat{H}^q\hat{\rho}^q\hat{\rho}^Q\right)_{PQ} - \left(\hat{\rho}^q\hat{\rho}^Q\hat{H}^q\right)_{PQ} \\ &= [\hat{H}^q,\hat{\rho}^q]\,f(Q,P),\end{aligned} \tag{S5}$$

where $f(Q,P)=\hat{\rho}^{Q}_{PQ}=\langle\langle PQ|\rho^{Q}\rangle\rangle$ is the classical distribution function of the subsystem $\hat{Q}$.

The second term in Eq. S3a

$$\begin{aligned}\left[\hat{H}^{Q},\hat{\rho}\right]_{PQ}&=\hat{\rho}^{q}\left[\hat{H}^{Q},\hat{\rho}^{Q}\right]_{PQ}\\&=\hat{\rho}^{q}\left(H^{Q}(Q,P)e^{\frac{i\hbar}{2}T}f(Q,P)-H^{Q}(Q,P)e^{-\frac{i\hbar}{2}T}f(Q,P)\right)\end{aligned}\tag{S6}$$

Here $H^{Q}(Q,P)=\hat{H}^{Q}_{PQ}=\langle\langle PQ|H^{Q}\rangle\rangle$ is classical Hamiltonian of the subsystem $\hat{Q}$, and operator $T=\frac{\overleftarrow{\partial}}{\partial Q}\frac{\overrightarrow{\partial}}{\partial P}-\frac{\overleftarrow{\partial}}{\partial P}\frac{\overrightarrow{\partial}}{\partial Q}$. We use Taylor series expansion for the exponents and neglect terms with $\hbar^{n}, n\geq 2$:

$$\left[\hat{H}^{Q},\hat{\rho}\right]_{PQ}=i\hbar\,\hat{\rho}^{q}\,\{H^{Q}(Q,P),f(Q,P)\}\tag{S7}$$

Poisson bracket $\{x,y\}=xTy$.

The third term in Eq. S3a

$$\begin{aligned}\left[\hat{H}^{qQ},\hat{\rho}\right]_{PQ}&=\left(\hat{H}^{qQ}\hat{\rho}^{q}\hat{\rho}^{Q}\right)_{PQ}-\left(\hat{\rho}^{q}\hat{\rho}^{Q}\hat{H}^{qQ}\right)_{PQ}\\&=\hat{H}^{qQ}(Q,P)e^{\frac{i\hbar}{2}T}f(Q,P)\hat{\rho}^{q}-\hat{\rho}^{q}f(Q,P)e^{\frac{i\hbar}{2}T}\hat{H}^{qQ}(Q,P).\end{aligned}\tag{S8}$$

Hamiltonian $\hat{H}^{qQ}(Q,P)=\hat{H}^{qQ}_{PQ}=\langle\langle PQ|H^{qQ}\rangle\rangle$ is an operator in $\hat{q}$-space and function Q and P coordinates of subsystem $\hat{Q}$. We use Taylor series expansion for the exponents and neglect terms with $\hbar^{n}, n\geq 2$:

$$\begin{aligned}\left[\hat{H}^{qQ},\hat{\rho}\right]_{PQ}&=\left[\hat{H}^{qQ}(Q,P),\hat{\rho}^{q}\right]f(Q,P)\\&+\frac{i\hbar}{2}\left(\left\{\hat{H}^{qQ}(Q,P),f(Q,P)\hat{\rho}^{q}\right\}-\left\{f(Q,P)\hat{\rho}^{q},\hat{H}^{qQ}(Q,P)\right\}\right)\end{aligned}\tag{S9}$$

By substituting Eqs. S5, S7, and S9 into Eq. S3a, we obtain the Liouville equation of motion in partial Wigner representation:

$$\begin{aligned}\frac{\partial\hat{\rho}^{q}}{\partial t}f(Q,P)+\hat{\rho}^{q}\frac{\partial f(Q,P)}{\partial t}&=\overbrace{-\frac{i}{\hbar}\left[\hat{H}^{q},\hat{\rho}^{q}\right]f(Q,P)}^{I}+\overbrace{\hat{\rho}^{q}\{H^{Q}(Q,P),f(Q,P)\}}^{II}\\&\overbrace{-\frac{i}{\hbar}\left[\hat{H}^{qQ}(Q,P),\hat{\rho}^{q}\right]f(Q,P)}^{III}\\&+\overbrace{\frac{1}{2}\left(\left\{\hat{H}^{qQ}(Q,P),f(Q,P)\hat{\rho}^{q}\right\}-\left\{f(Q,P)\hat{\rho}^{q},\hat{H}^{qQ}(Q,P)\right\}\right)}^{IV}.\end{aligned}\tag{S10}$$

Terms I and II in this equation describe the evolution of the quantum and classical subsystems without interaction. Term III describes the influence on the quantum subsystem by the classical degrees of freedom. The last term IV reflects the influence on the evolution of classical subsystem by quantum

degrees of freedom. Equation S10 is identical to the previously derived mixed quantum-classical equation of motion[1].

In Eq. S10, we neglect the last Term IV, i.e., we assume that quantum subsystems do not influence the evolution of the classical bath. The same approximation is used in 1D and 2D spectroscopy calculations using the frequency map approach[2]. One can expect a violation of this approximation for systems with fast energy relaxation from quantum degrees of freedom. Nevertheless, simulations using the frequency map approach show very good agreement with experiments for absorption, Raman and 2D IR spectroscopy, even for O-H stretch vibration of liquid water[3], which has very fast energy dissipation (≈100s of fs[4]). Hence, this approximation can be reasonable for a wide range of vibrational oscillators.

We can group terms describing the evolution of quantum and classical subsystems and transform Eq. S10 into a system of two coupled equations:

$$\frac{\partial \hat{\rho}^q}{\partial t} = -\frac{i}{\hbar}\left[\hat{H}^q, \hat{\rho}^q\right] - \frac{i}{\hbar}\left[\hat{H}^{qQ}(Q,P), \hat{\rho}^q\right], \tag{S11}$$

$$\frac{\partial f(Q,P)}{\partial t} = \{H^Q(Q,P), f(Q,P)\}. \tag{S12}$$

In this model, the evolution of the quantum subsystem is affected by interaction with the bath, which evolves independently.

The equation of motion of the bath can be written using the classical Liouville operator[5]:

$$\frac{\partial f(Q,P)}{\partial t} = -iL^Q(Q,P)f(Q,P). \tag{S13}$$

The solution to this equation reads:

$$\begin{aligned} f(Q,P,t) &= e^{-iL^Q(Q,P)\tau} f(Q,P,t-\tau) \\ &= U^Q(\tau) f(Q,P,t-\tau) \end{aligned} \tag{S14}$$

We can write Eq. S11 using Liouville space notations:

$$\frac{\partial |\rho^q(t)\rangle\rangle}{\partial t} = -\frac{i}{\hbar}\left(L^q + L^{qQ}(Q,P)\right)|\rho^q(t)\rangle\rangle. \tag{S15}$$

The Liouville superoperator describing the interaction of the subsystems parametrically depends on classical coordinates.

We consider a trajectory of classical bath described by functions $Q(t)$ and $P(t)$. For this trajectory, the solution of Eq. S15 is

$$|\rho^q(t)\rangle\rangle = e^{-\frac{i}{\hbar}\int_{t_0}^{t} dt'\left(L^q + L^{qQ}\left(Q(t'),P(t')\right)\right)} |\rho^q(t_0)\rangle\rangle, \tag{S16}$$

Where $|\rho^q(t_0)\rangle\rangle$ is the initial state of the quantum subsystem. Thus, the state of the quantum subsystem at any time depends on the initial state $|\rho^q(t_0)\rangle\rangle$ and trajectory $Q(t)$ and $P(t)$ of the classical bath. Because classical motion is deterministic and coordinates Q and P at any time define the full trajectory of the system, for a given trajectory, we can consider $|\rho^q(t)\rangle\rangle$ to depend parametrically on the coordinates of the classical bath at the same moment of time:

$$|\rho^q(Q(t),P(t),t)\rangle\rangle = e^{-\frac{i}{\hbar}\int_{t_0}^{t} dt'\left(L^q + L^{qQ}\left(Q(t'),P(t')\right)\right)} |\rho^q(Q(t_0),P(t_0),t_0)\rangle\rangle. \tag{S17}$$

Therefore, for the entire phase space of the classical bath, we can write:

$$|\rho^q(Q,P,t)\rangle\rangle = U^q(Q,P,\tau)\, \left|\rho^q\left(Q(t-\tau),P(t-\tau),(t-\tau)\right)\right\rangle\Big\rangle, \tag{S18}$$

where evolution superoperator of quantum subsystem

$$U^q(Q,P,\tau) = e^{-\frac{i}{\hbar}\int_{t-\tau}^{t} dt'\left(L^q + L^{qQ}\left(Q(t'),P(t')\right)\right)}. \tag{S19}$$

The density matrix of the entire system at time t in partial Wigner representation is given by:

$$\begin{aligned} \langle\langle PQ|\rho(t)\rangle\rangle &= |\rho^q(Q,P,t)\rangle\rangle f(Q,P,t) \\ &= \langle\langle PQ|U(\tau)|\rho(t-\tau)\rangle\rangle \\ &= U^Q(\tau) f(Q,P,t-\tau) U^q(Q,P,\tau)\, \left|\rho^q\left(Q(t-\tau),P(t-\tau),(t-\tau)\right)\right\rangle\Big\rangle \\ &= U^q(Q,P,\tau) U^Q(\tau) \langle\langle PQ|\rho(t-\tau)\rangle\rangle \end{aligned} \tag{S20}$$

B. Mixed quantum-classical response function in 2D TIRV spectroscopy

Using the quantum formalism, the response function of a sample measured in 2D TIRV spectroscopy is given by[6–8]:

$$S^{(3)}(t_2,t_1) = \left(\frac{i}{\hbar}\right)^2 \langle\langle \Pi|\, U(t_2)\, M\, U(t_1)\, M|\rho(-\infty)\rangle\rangle, \tag{S21}$$

where $M = [\hat{\mu}^{qQ}, \dots]$ is dipole moment superoperator ($\hat{\mu}^{qQ}$ is dipole moment operator of the system), U is the evolution superoperator and $\widehat{\Pi}$ is the polarizability operator. We use identity superoperator $I = \iint dQdP |PQ\rangle\rangle\langle\langle PQ|$ to write response function in partial Wigner representation[9]:

$$S^{(3)}(t_2, t_1) = \left(\frac{i}{\hbar}\right)^2 \iint dQdP \,\langle\langle \Pi | PQ \rangle\rangle \langle\langle PQ | U(t_2)\, M\, U(t_1)\, M | \rho(-\infty) \rangle\rangle. \tag{S22}$$

The polarizability

$$\langle\langle \Pi | PQ \rangle\rangle = \langle\langle \Pi^q(Q, P)| \tag{S23}$$

is an operator in $\hat{q}$-space, which parametrically depends on coordinates Q and P of the bath.

Using Eq. S20, the second part under the integral in Eq. S22:

$$\langle\langle PQ | U(t_2)\, M\, U(t_1)\, M | \rho(-\infty) \rangle\rangle = U^q(Q, P, t_2) U^Q(t_2) \langle\langle PQ | M\, U(t_1) M | \rho(-\infty) \rangle\rangle. \tag{S24}$$

Thus, the response function

$$\begin{aligned} S^{(3)}(t_2, t_1) = \left(\frac{i}{\hbar}\right)^2 \iint dQdP \langle\langle \Pi^q(Q, P)|\, U^q(Q, P, t_2) U^Q(t_2) \\ \times \langle\langle PQ | M\, U(t_1) M | \rho(-\infty) \rangle\rangle. \end{aligned} \tag{S25}$$

By substituting another identity superoperator we obtain:

$$\begin{aligned} S^{(3)}(t_2, t_1) = \left(\frac{i}{\hbar}\right)^2 \iint dQdP \langle\langle \Pi^q(Q, P)|\, U^q(Q, P, t_2) U^Q(t_2) \\ \times \iint dQ'dP' \,\langle\langle PQ | M U(t_1) | P'Q' \rangle\rangle \langle\langle P'Q' | M | \rho(-\infty) \rangle\rangle \end{aligned} \tag{S26}$$

The superoperator $M = [\hat{\mu}^{qQ}, \dots]$ is composed of operators in both $\hat{q}$ and $\hat{Q}$ spaces. However, because the THz field is in resonance with low-frequency motion, we assume that in the first interaction electromagnetic field acts only on the subsystem $\hat{Q}$ and dipole moment operator $\hat{\mu}^{qQ} = \hat{\mu}^{Q}_{q_0}$ (q_0 is the equilibrium position of quantum degrees of freedom). In this approximation:

$$\langle\langle P'Q'|M|\rho(-\infty)\rangle\rangle = |\rho^q(-\infty)\rangle\rangle\left[\hat{\mu}^Q_{q_0}, \hat{\rho}^Q(-\infty)\right]_{P'Q'}$$
$$= |\rho^q(-\infty)\rangle\rangle\left(\left(\hat{\mu}^Q_{q_0}\hat{\rho}^Q(-\infty)\right)_{P'Q'} - \left(\hat{\rho}^Q(-\infty)\hat{\mu}^Q_{q_0}\right)_{P'Q'}\right)$$
$$= |\rho^q(-\infty)\rangle\rangle\left(\mu^c_{q_0}(Q')e^{\frac{i\hbar}{2}T}f(Q',P',-\infty) - \mu^c_{q_0}(Q')e^{-\frac{i\hbar}{2}T}f(Q',P',-\infty)\right) \tag{S27}$$

By using the Taylor series of the exponents and neglecting terms with $\hbar^n, n \geq 2$, we obtain:

$$\langle\langle P'Q'|M|\rho(-\infty)\rangle\rangle = i\hbar|\rho^q(-\infty)\rangle\rangle\{\mu^c_{q_0}(Q'), f(Q',P',-\infty)\}. \tag{S28}$$

By substituting Eq. S28 and one more identity superoperator into Eq. S26, we obtain:

$$S^{(3)}(t_2,t_1) = -\frac{i}{\hbar}\iint dQdP\langle\langle\Pi^q(Q,P)|\, U^q(Q,P,t_2)U^Q(t_2) \times \iint dQ'dP' \iint dQ''dP''\, \langle\langle PQ|M|P''Q''\rangle\rangle \times \langle\langle P''Q''|U(t_1)|P'Q'\rangle\rangle|\rho^q(-\infty)\rangle\rangle\{\mu^c_{q_0}(Q'), f(Q',P',-\infty)\} \tag{S29}$$

Using Eq. S20 the term

$$\langle\langle P''Q''|U(t_1)|P'Q'\rangle\rangle|\rho^q(-\infty)\rangle\rangle = U^q(Q'',P'',t_1)U^Q(t_1)|\rho^q(Q''(-\infty),P''(-\infty),-\infty)\rangle\rangle \times \delta(Q'-Q'')\delta(P'-P''). \tag{S30}$$

Initially, the quantum subsystem is in thermal equilibrium, therefore:

$$U^q(Q'',P'',t_1)|\rho^q(Q''(-\infty),P''(-\infty),-\infty)\rangle\rangle = |\rho^q(Q'',P'',-\infty)\rangle\rangle \tag{S31}$$

The response function

$$S^{(3)}(t_2,t_1) = -\frac{i}{\hbar}\iint dQdP\langle\langle\Pi^q(Q,P)|\, U^q(Q,P,t_2)U^Q(t_2) \times \iint dQ'dP'\, \langle\langle PQ|M|P'Q'\rangle\rangle|\rho^q(Q',P',-\infty)\rangle\rangle \times U^Q(t_1)\{\mu^c_{q_0}(Q'), f(Q',P',-\infty)\} \tag{S32}$$

We consider the term containing the dipole moment:

$$\left\langle \langle PQ|M|P'Q'\rangle \middle| \rho^q(Q',P',-\infty)\rangle \right\rangle = \left[\hat{\mu}^{qQ}, \hat{\rho}^{P'Q'}\ \hat{\rho}^q(Q',P',-\infty)\right]_{PQ}$$
$$= \left(\hat{\mu}^{qQ}_{PQ}\, e^{\frac{i\hbar}{2}T}\ \hat{\rho}^{P'Q'}_{PQ}\hat{\rho}^q(Q',P',-\infty) - \hat{\rho}^q(Q',P',-\infty)\hat{\rho}^{P'Q'}_{PQ}\, e^{\frac{i\hbar}{2}T}\hat{\mu}^{qQ}_{PQ}\ \right). \qquad (S33)$$

$\hat{\mu}^{qQ}_{PQ} = \hat{\mu}^q(Q,P)$ is transition dipole moment operator of the quantum subsystem parametrically depending on bath degrees of freedom, and $\hat{\rho}^{P'Q'}_{PQ} = \langle\langle PQ|P'Q'\rangle\rangle = \delta(Q'-Q)\delta(P'-P)$.

By taking the Taylor series for exponents and neglecting terms with $\hbar^n, n \geq 1$, we obtain:

$$\left\langle \langle PQ|M|P'Q'\rangle \middle| \rho^q(Q',P',-\infty)\rangle \right\rangle = [\hat{\mu}^q(Q,P), \hat{\rho}^q(Q',P',-\infty)]\delta(Q'-Q)\delta(P'-P). \qquad (S34)$$

By substituting Eq. S34 into Eq. S32, we obtain:

$$S^{(3)}(t_2,t_1) = -\frac{i}{\hbar}\iint dQdP\langle\langle \Pi^q(Q,P)|\, U^q(Q,P,t_2)U^Q(t_2)[\hat{\mu}^q(Q,P), \hat{\rho}^q(Q,P,-\infty)]$$
$$\times U^Q(t_1)\{\mu^c_{q_0}(Q), f(Q,P,-\infty)\}. \qquad (S35)$$

Because $f(Q,P,-\infty)$ is an equilibrium distribution function, we can write[10]:

$$\{\mu^c_{q_0}(Q), f(Q,P,-\infty)\} = -\frac{1}{k_\mathrm{B}T}\dot{\mu}^c_{q_0}(Q)f(Q,P,-\infty). \qquad (S36)$$

Then:

$$U^Q(t_1)\left(-\frac{1}{k_BT}\dot{\mu}^c_{q_0}(Q)f(Q,P,-\infty)\right) = -\frac{1}{k_\mathrm{B}T}\dot{\mu}^c_{q_0}\big(Q(-t_1)\big)f(Q,P,-\infty). \qquad (S37)$$

Equation S35 takes the form:

$$S^{(3)}(t_2,t_1) = \frac{1}{k_\mathrm{B}T}\frac{i}{\hbar}\iint dQdP\langle\langle \Pi^q(Q,P)|\, U^q(Q,P,t_2)U^Q(t_2)[\hat{\mu}^q(Q,P), \hat{\rho}^q(Q,P,-\infty)]$$
$$\times \dot{\mu}^c_{q_0}\big(Q(-t_1)\big)f(Q,P,-\infty). \qquad (S38)$$

We assume that the quantum subsystem is initially in the ground state and $\hat{\rho}^q(Q,P,-\infty) = |g\rangle\langle g|$. We consider excitation to state $|f\rangle\langle g|$, which generates the signal in the first and second quadrants of the 2D TIRV spectrum:

$$\begin{aligned}[\hat{\mu}^q(Q,P), |g\rangle\langle g|] &= |f\rangle\langle g|\mu^q_{fg}(Q,P) \\ &= |f,g\rangle\rangle\mu^q_{fg}(Q,P).\end{aligned} \tag{S39}$$

Here $\mu^q_{fg}(Q,P) = \langle f|\hat{\mu}^q(Q,P)|g\rangle$. The response function for this excitation pathway reads:

$$\begin{aligned}S^{(3)}(t_2,t_1) = \frac{1}{k_\mathrm{B}T}\frac{i}{\hbar}\iint dQdP\big\langle\langle\Pi^q(Q,P)|\, U^q(Q,P,t_2)|f,g\rangle\big\rangle \\ \times U^Q(t_2)\mu^q_{fg}(Q,P)\dot{\mu}^c_{q_0}\big(Q(-t_1)\big)f(Q,P,-\infty).\end{aligned} \tag{S40}$$

This equation can be written in the final form:

$$\begin{aligned}S^{(3)}(t_2,t_1) = \frac{1}{k_\mathrm{B}T}\frac{i}{\hbar}\iint dQdP\big\langle\langle\Pi^q(Q,P)|\, U^q(Q,P,t_2)|f,g\rangle\big\rangle \\ \times\, \mu^q_{fg}\big(Q(-t_2),P(-t_2)\big)\dot{\mu}^c_{q_0}\big(Q(-t_1-t_2)\big)f(Q,P,-\infty).\end{aligned} \tag{S41}$$

For multiple excited states $|f\rangle$ of the quantum subsystem, the full response function in the first and second quadrants is given by the sum of individual excitation pathways:

$$\begin{aligned}S^{(3)}(t_2,t_1) = \frac{1}{k_\mathrm{B}T}\frac{i}{\hbar}\sum_f\iint dQdP\big\langle\langle\Pi^q(Q,P)|\, U^q(Q,P,t_2)|f,g\rangle\big\rangle \\ \times\, \mu^q_{fg}\big(Q(-t_2),P(-t_2)\big)\dot{\mu}^c_{q_0}\big(Q(-t_1-t_2)\big)f(Q,P,-\infty)\end{aligned} \tag{S42}$$

It is straightforward to write scalar Eqs. S41 and S42 in tensor form.

Parameters of mid-infrared vibrational resonances of DMSO in Raman spectrum.

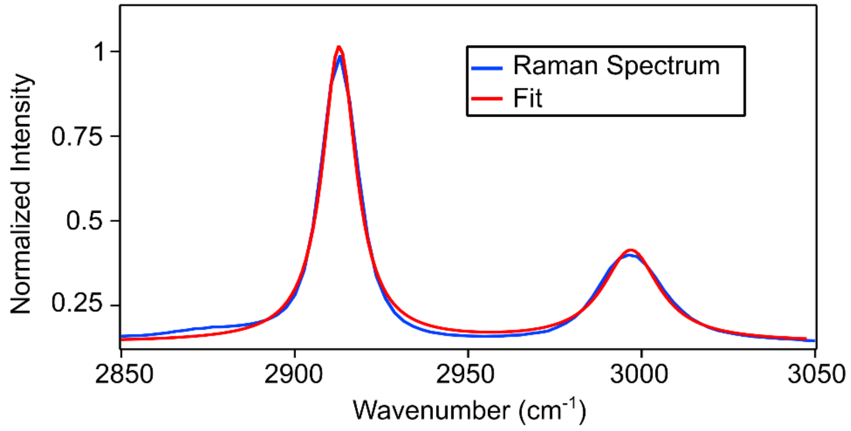

FIG. S2. Lorentzian fits to the HFMs in the DMSO Raman spectrum.

TABLE S1. The fitting parameters of DMSO Raman spectrum.

	Centre wavenumber (cm^{-1})	Area under the curve (normalized)
Symmetric C-H stretch	2913	1
Asymmetric C-H stretch	2997	0.51

Parameters of THz and mid-infrared vibrational resonances of DMSO in absorption spectrum.

Figure S3 shows the fitting of LFMs and HFMs in DMSO absorption spectrum to a superposition of Lorentzian (LFM2, LFM3, symmetric and asymmetric CH_3 stretch) and Gaussian (LFM1) functions. The fitting parameters are summarized in Table S2.

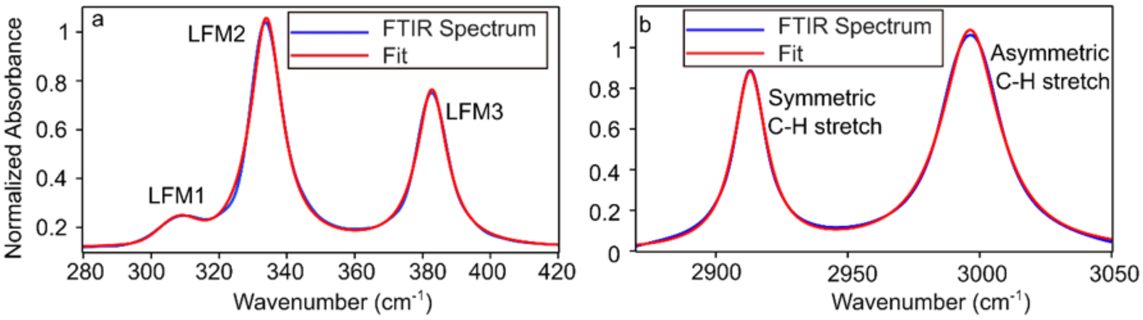

FIG. S3. Fits to (a) LFMs and (b) HFMs in the DMSO absorption spectrum.

TABLE S2. The fitting parameters of the DMSO absorption spectrum.

	Centre wavenumber (cm^{-1})	Area under the curve (a.u.)
LFM 1	308	1.23
LFM 2	334	16.75
LFM 3	383	11.1
Symmetric C-H stretch	2913	10.33
Asymmetric C-H stretch	2996	22.8

First-order response of low-frequency modes of DMSO

Figure S4 shows the simulated spectrum of the first-order response of DMSO low-frequency modes. The spectrum is obtained by Fourier transformation of the time-domain response function $S^{(1)}_{\mathrm{DMSO}}$, which is given by[9]:

$$S^{(1)}_{\mathrm{DMSO}}(t) = \frac{1}{k_{\mathrm{B}}T}\iint dQdP\mu^{c}_{q_0}\big(Q(t)\big)\dot{\mu}^{c}_{q_0}\big(Q(0)\big).$$

The dipole moment of the LFMs is given by:

$$\mu^{c}_{q_0}(Q) = \sum_{n} Z_n Q^0_n \sin\left(\int_0^t (\Omega_n + \delta\Omega_n(\tau))\, d\tau + \varphi_n\right).$$

The calculations are performed using parameters $Z_n Q^0_n$, Ω_n and $\delta\Omega_n$ of symmetric CH_3 stretch mode determined in the 2D TIRV model with mechanical coupling (Table 1). In an additional calculation (blue line in Fig. S4) we assume $Z_4 Q^0_4 = 0$. Thus, the difference between red and blue spectra shows the absorbance of the $n = 4$ mode. These results demonstrate that the parameters of the 2D TIRV model are consistent with the DMSO absorption spectrum in Fig. S1, i.e., absorption of resonance $n = 4$ is weak and not pronounced.

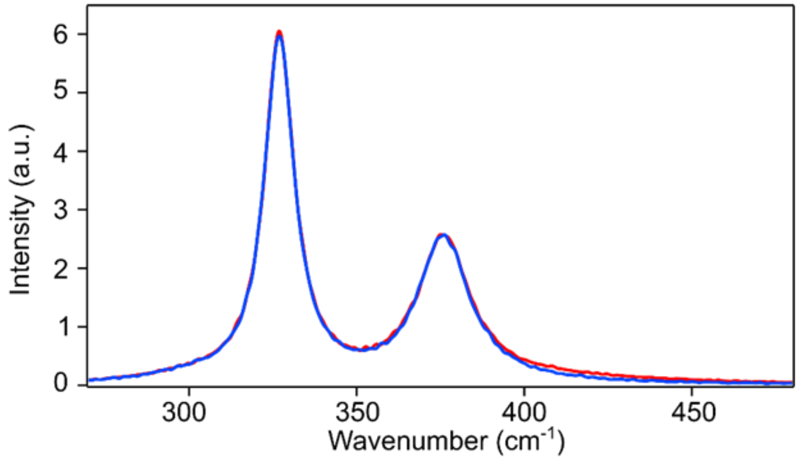

FIG. S4. The red line shows the imaginary part of the first-order response function of DMSO in the terahertz range. It is calculated using the same transition dipole moments and frequency fluctuations as in the 2D TIRV model (with mechanical coupling). The blue line shows a similar simulation but assumes zero dipole moment for the n=4 mode.

Comparison of experimental and calculated 2D TIRV spectra of DMSO

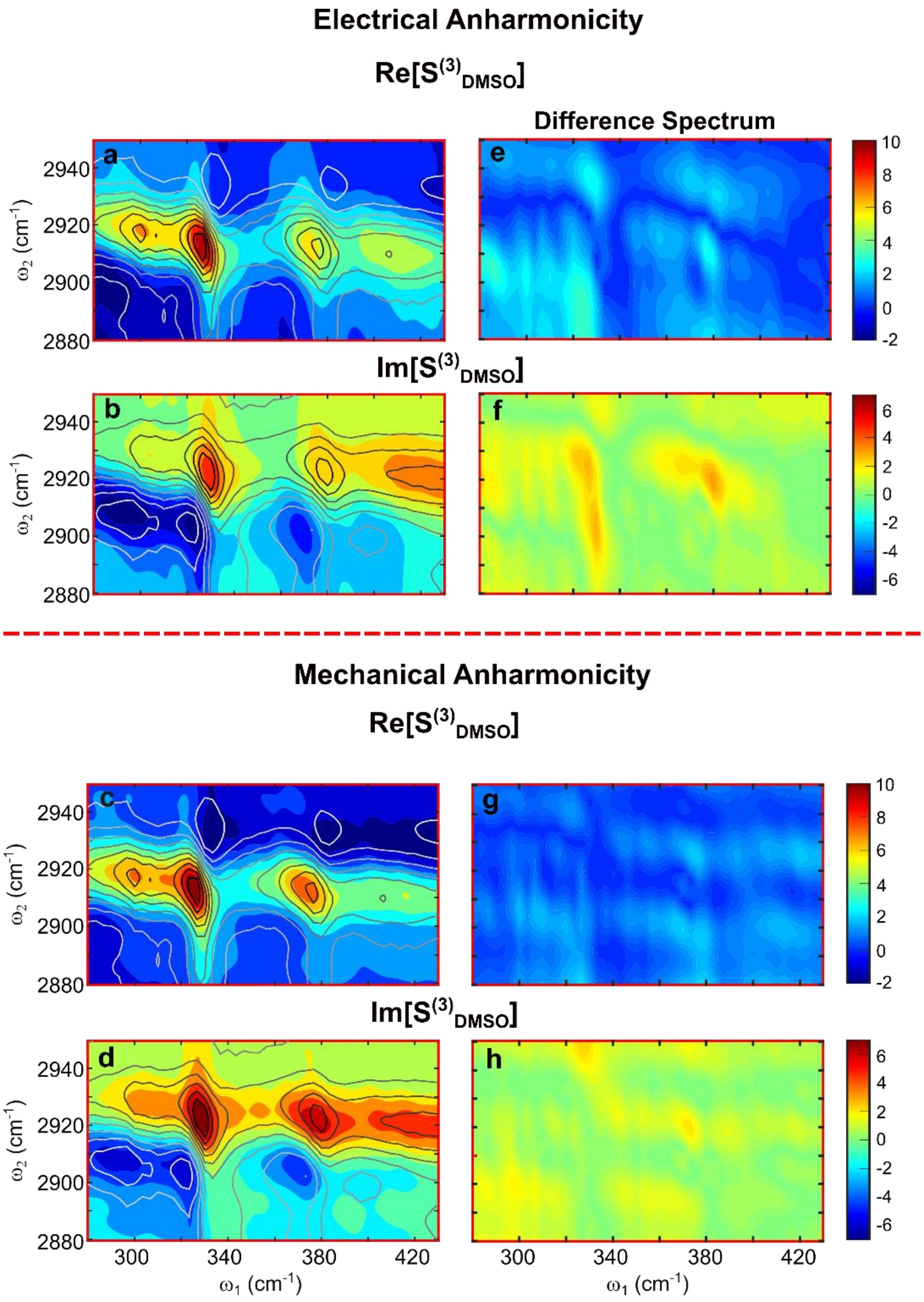

FIG. S5. Comparison of experimental data and theoretical models. In (a), (b), (c) and (d) calculated and experimental spectra are shown by colored and line contours, respectively. (e), (f), (g) and (h) show corresponding difference spectra (calculated spectra are subtracted from experimental).

Comparison of lineshapes produced by electrical and mechanical anharmonicity

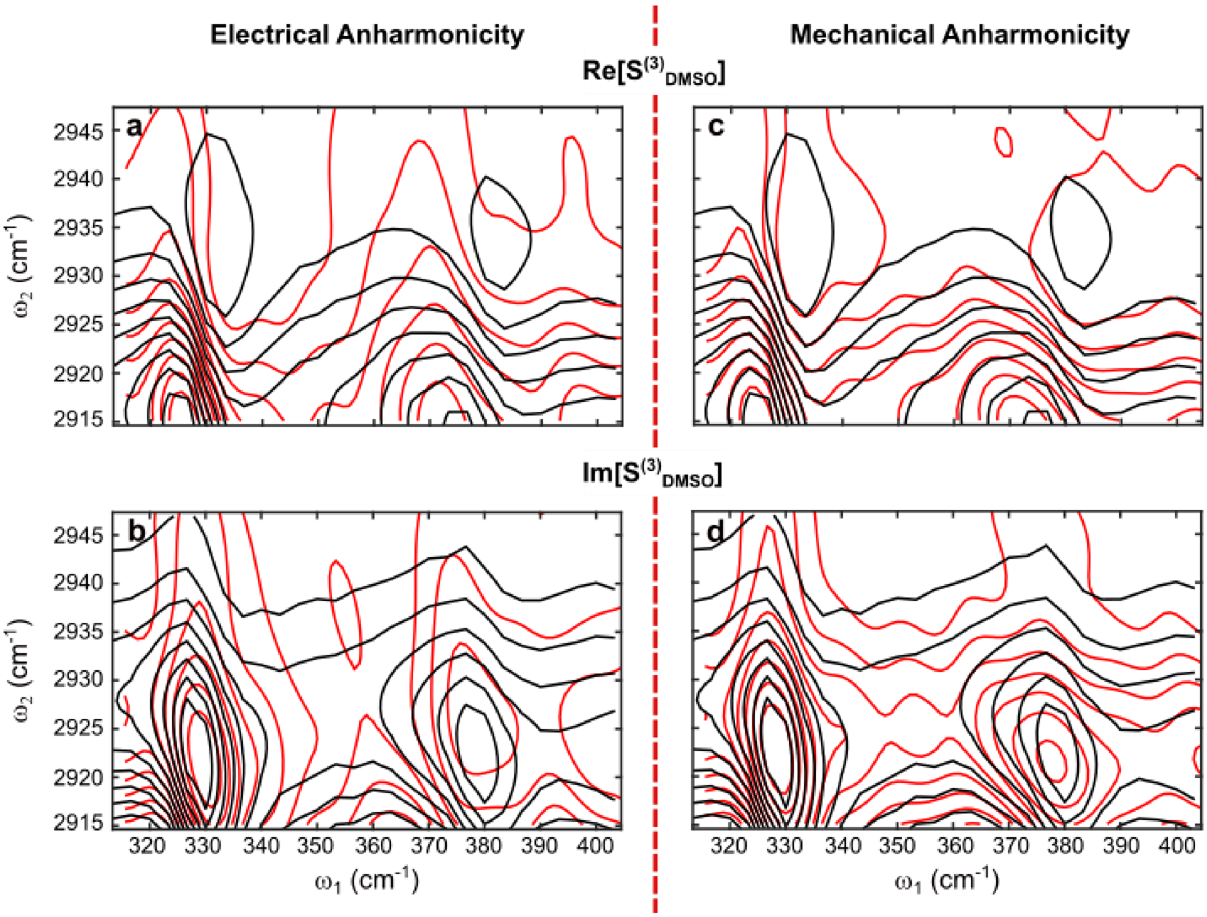

FIG. S6. Comparison of theoretical and experimental spectra of DMSO. Red contour lines show real (a,c) and imaginary (b,d) parts of the 2D TIRV response function calculated for the case of electrical (a,b) and mechanical (c,d) anharmonicity. Black contour lines show the measured spectrum.

Comparison of vertical cuts though a single resonance

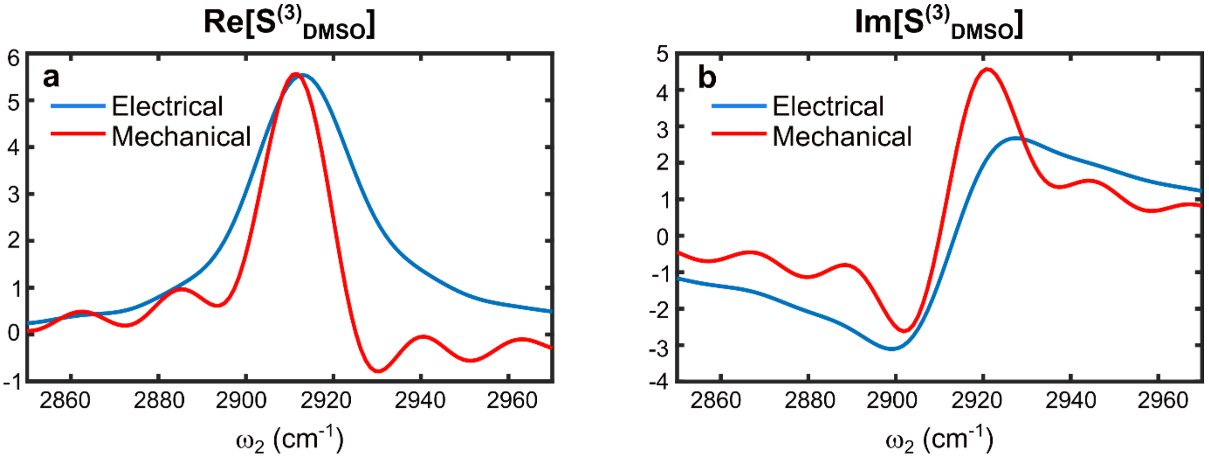

FIG. S7. Comparison of vertical cuts through a single 2D TIRV resonance for electrical (blue) and mechanical (red) anharmonicities. (a) and (b) show real and imaginary parts, respectively.

Supplemental References

(1) Kapral, R.; Ciccotti, G. Mixed Quantum-Classical Dynamics. *J. Chem. Phys.* **1999**, *110* (18), 8919–8929.

(2) Jansen, T. la C.; Saito, S.; Jeon, J.; Cho, M. Theory of Coherent Two-Dimensional Vibrational Spectroscopy. *J. Chem. Phys.* **2019**, *150* (10), 100901.

(3) Auer, B.; Kumar, R.; Schmidt, J. R.; Skinner, J. L. Hydrogen Bonding and Raman, IR, and 2D-IR Spectroscopy of Dilute HOD in Liquid D_2O. *Proc. Natl. Acad. Sci.* **2007**, *104* (36), 14215–14220.

(4) Woutersen, S.; Emmerichs, U.; Nienhuys, H.-K.; Bakker, H. J. Anomalous Temperature Dependence of Vibrational Lifetimes in Water and Ice. *Phys. Rev. Lett.* **1998**, *81* (5), 1106–1109.

(5) Zwanzig, R. Ensemble Method in the Theory of Irreversibility. *J. Chem. Phys.* **1960**, *33* (5), 1338–1341.

(6) Ito, H.; Jo, J.-Y.; Tanimura, Y. Notes on Simulating Two-Dimensional Raman and Terahertz-Raman Signals with a Full Molecular Dynamics Simulation Approach. *Struct. Dyn.* **2015**, *2* (5), 54102.

(7) Tanimura, Y.; Mukamel, S. Two-dimensional Femtosecond Vibrational Spectroscopy of Liquids. *J. Chem. Phys.* **1993**, *99* (12), 9496–9511.

(8) Ikeda, T.; Ito, H.; Tanimura, Y. Analysis of 2D THz-Raman Spectroscopy Using a Non-Markovian Brownian Oscillator Model with Nonlinear System-Bath Interactions. *J. Chem. Phys.* **2015**, *142* (21), 212421.

(9) Mukamel, S. *Principles of Nonlinear Optical Spectroscopy*; Oxford University Press: New York, 1995.

(10) Williams, R. B.; Loring, R. F. Classical Mechanical Photon Echo of a Solvated Anharmonic Vibration. *J. Chem. Phys.* **2000**, *113* (5), 1932–1941.
