# Supplementary material for: Experimental Access to Mode-Specific Coupling between Quantum Molecular Vibrations and Classical Bath Modes
Source: J Phys Chem Lett. 2023 Sep 20;14(38):8630–7. doi: 10.1021/acs.jpclett.3c01974 (PMC10544034; doi:10.1021/acs.jpclett.3c01974)
Supplement: Supplementary file 2 — jz3c01974_si_002.pdf [file jz3c01974_si_002.pdf]

jz-2023-01974q.R1

Name: Peer Review Information for "Experimental Access to Mode-Specific Coupling between Quantum Molecular Vibrations and Classical Bath Modes"

First Round of Reviewer Comments

Reviewer: 1

Comments to the Author

This is an excellent paper, likely to become a classic. The experimental and theoretical work is novel and important. The science is rigorous, and the presentation is clear. The paper will be of interest to the broad readership of JPCL. The only thing I would suggest is changing the title to more accurately describe the importance of this work. Currently, a reader has no idea of what the paper actually contains.

Reviewer: 2

Comments to the Author

In this paper the authors describe a model to extract coupling between a high frequency vibrational mode (quantum description) and low-frequency vibrational modes (classical description) in a neat liquid, dimethylsulfoxide using a newly developed spectroscopic technique employing a sequence of THz and IR femtosecond pulses, and a non resonant visible picosecond pulse.

This reviewer agrees that the technique is promising and the ability to extract anharmonic couplings between low and high frequency vibrational modes will likely impact all areas of physical chemistry.

However, the scholarly presentation is poor, both in terms of writing and the figures. This makes it very difficult for the reader to understand the significance, and novelty of the work. In addition, the figure presentation makes it very difficult to determine the quality of the simulation/fits and their comparison to the data. Key pieces of experimental information is missing.

For these reasons, this reviewer does not recommend publication in JPC Letters.

Author's Response to Peer Review Comments:

Reply to reviewers' comments and revisions to the manuscript: all revisions are highlighted in the Supporting Information for Review Only.

Reviewer #1:

Recommendation: This paper represents a significant new contribution and should be published as is.

Comments:
This is an excellent paper, likely to become a classic. The experimental and theoretical work is novel and important. The science is rigorous, and the presentation is clear. The paper will be of interest to the broad readership of JPCL. The only thing I would suggest is changing the title to more accurately describe the importance of this work. Currently, a reader has no idea of what the paper actually contains.

> We appreciate the very positive comment. We have addressed the Reviewer's remark by changing title of the revised manuscript to: "Experimental Access to Mode-Specific Coupling between Quantum Molecular Vibrations and Classical Bath Modes".

Reviewer #2:

Recommendation: This paper is not recommended because the material is not appropriate for The Journal of Physical Chemistry Letters.

Comments:
In this paper the authors describe a model to extract coupling between a high frequency vibrational mode (quantum description) and low-frequency vibrational modes (classical description) in a neat liquid, dimethylsulfoxide using a newly developed spectroscopic technique employing a sequence of THz and IR femtosecond pulses, and a non resonant visible picosecond pulse.

This reviewer agrees that the technique is promising and the ability to extract anharmonic couplings between low and high frequency vibrational modes will likely impact all areas of physical chemistry.

We appreciate the Reviewer's remarks and the positive comment regarding the potential of the experimental and theoretical methods reported in the present work.

However, the scholarly presentation is poor, both in terms of writing and the figures. This makes it very difficult for the reader to understand the significance, and novelty of the work. In addition, the figure presentation makes it very difficult to determine the quality of the simulation/fits and their comparison to the data. Key pieces of experimental information is missing.

For these reasons, this reviewer does not recommend publication in JPC Letters.

Because our experimental and theoretical methods are complicated and new, we paid special attention to making their discussion clear when preparing the original manuscript. We are puzzled by the Reviewer's remark, also in view of the very positive evaluation of the presentation by Reviewer 1. We would appreciate a more detailed comment from Reviewer 2 that would have allowed us to better address potential sources of confusion. In the present revision, we have tried our best to address the Reviewer's concerns and improve the clarity of our manuscript by making the following changes:

1. In the revised manuscript, we added the energy level diagrams of a 2D TIRV excitation pathway in Figure 1. We believe that this diagram can be helpful for the readers' understanding of 2D TIRV spectroscopy principles.
2. We have added a color bar to the 2D TIRV spectrum of DMSO in Figure 1c in the revised manuscript.
3. We have added color bars to Figure 3 in the revised manuscript.
4. We have changed the color scheme for the contour lines that show experimental data in Figure 3. In the original manuscript, we used black lines. To ease comparison with the theoretical spectrum, we have used grayscale contours and added corresponding grayscale bar in Figure 3 in the revised manuscript.
5. For better demonstrating agreement between calculations and experiment we have added difference spectra in Fig. S5 in the Supporting Information.

We have also added Fig. S6 in the Supporting Information to easily compare the experimental and theoretical lineshapes. In this figure, we zoom into the $\omega_1 = 315 - 405$ cm^{-1}, $\omega_2 = 2915 - 2947$ cm^{-1} frequency range of 2D TIRV spectrum. The lineshapes produced by electrical and mechanical anharmonicities have qualitative difference, which stems from uncorrelated/correlated frequency fluctuations of HFMs and LFMs, respectively. To improve the discussion of the origin of the lineshape difference between electrical and mechanical anharmonicity, we have added Figure 4 to the revised manuscript. This figure was part of the Supporting Information in the original submission. We have also added new Fig. S7 in the Supporting Information that shows vertical cuts through single 2D TIRV resonance produced by electrical and mechanical anharmonicities. We have modified the discussion correspondingly on page 12 of the revised manuscript:

> "Figures 3c,d show excellent agreement between this model and the experiment (Fig. S5 in Supporting Information shows the difference spectrum, and the model parameters are listed in Table I). The lineshapes match exceptionally well for both the real and imaginary parts of the response (see also Fig. S6 in Supporting Information). Note that lineshapes produced by mechanical and electrical anharmonicities are qualitatively different, and this difference is rooted in the correlation of frequency fluctuations. In the computational model, HFMs and LFMs of DMSO are always broadened homogeneously and their individual lineshapes are given by Lorentzian functions, in agreement with the absorption spectrum (Fig. S3 in Supporting Information). With electrical HFM-LFM anharmonicity, the frequency fluctuations of CH_3 stretch vibrations and bright LFMs are not correlated. In this case, a single resonance in the 2D TIRV spectrum has lineshape symmetric with respect to its center (Fig. 4a,b). This lineshape is identical to the lineshape of homogeneously broadened vibrational resonances with uncorrelated frequency fluctuations in 2D IR spectra[10,29]. The lack of frequency fluctuation correlations results in symmetric Lorentzian profile of a vertical cut taken through the resonance (Fig. S7 in Supporting Information)[10], at odds with experimental lineshape. In contrast, the correlation of frequency fluctuations produced by mechanical coupling causes asymmetry in the lineshape of individual 2D TIRV peaks (Fig. 4c,d, Fig. S7 in Supporting Information). This results in an asymmetry in the overall spectrum in Fig. 3 (see also Fig. S6 in Supporting Information), in agreement with experimental lineshape."

6. We have added the following Methods section, providing experimental details on page 14 of the revised manuscript. This discussion was part of the Supporting information in the original submission.

> "We use femtosecond amplified Ti:sapphire laser system (Astrella, Coherent) with a repetition rate of 1 kHz, central wavelength of 800 nm and full width at half maximum (FWHM) of ~60 nm. The output of the laser is

split into three beams to generate broadband terahertz (THz), broadband infrared (IR), and narrowband visible (VIS) pulses. THz pulse is produced from a laser beam with an energy of about 1.1 mJ/pulse by two-color mixing in air plasma[40]. IR pulse is produced using commercial TOPAS (traveling wave optical parametric amplification of superfluorescence) coupled to NDFG (non-collinear difference frequency generation) unit. The TOPAS is pumped by a laser beam with ≈ 1 mJ/pulse energy. VIS pulse (FWHM ≈ 10 cm-1) is produced using a 4f pulse shaper from laser beam with ≈ 0.8 mJ/pulse energy. Local oscillator (LO) pulse is generated in a displaced Sagnac interferometer by sum frequency mixing of IR and VIS fields in a type 1 BBO crystal (10 μm thickness, Newlight Photonics Inc.). All the pulses are focused and overlapped at the sample to generate the third-order signal. After the sample, signal and LO are collimated and directed to a spectrometer (SpectraPro HRS-300, Princeton Instruments) equipped with an EMCCD detector (Newton 971, Andor). We use parallel polarization of THz, IR, VIS, and signal fields. Energies of IR and VIS pulses at the sample are about 1.5 μJ/pulse and 4.5 μJ/pulse, respectively. More details on the experimental setup, methods of data acquisition, and processing are provided elsewhere[17].

Time domain data are acquired by varying the time delay between THz and IR/VIS pulse pair in steps of 10 fs using a motorized delay stage (V-551.2B, Physik Instrumente (PI)). The total time intervals of scans for DMSO and SiN_x are 7.33 ps and 1.33 ps, respectively, with the same start position. It takes around 25 (5) minutes to acquire one spectrum of DMSO (SiN_x).

The front window of the DMSO sample cell is a 50 nm thick low-stress SiN_x membrane (NX5050A, Norcada) with 0.5 mm × 0.5 mm aperture (in 5mm×5mm, 200 μm thick silicon frame). The back window is 2 mm thick CaF_2 window. Front and back windows are separated by a 1 mm thick Viton o-ring.”
